# Accuracy and limitations of continuous glucose monitoring using spectroscopy in critically ill patients

**DOI:** 10.1186/2110-5820-4-8

**Published:** 2014-03-06

**Authors:** Roosmarijn TM van Hooijdonk, Tineke Winters, Johan C Fischer, Edmée C van Dongen-Lases, James S Krinsley, Jean-Charles Preiser, Marcus J Schultz

**Affiliations:** 1 Department of Intensive Care, Academic Medical Center, University of Amsterdam, Room, Meibergdreef 9, 1105 AZ, Amsterdam, the Netherlands; 2 Department of Clinical Chemistry, Academic Medical Center, University of Amsterdam, Amsterdam, the Netherlands; 3 Division of Critical Care, Stamford Hospital, Columbia University College of Physicians and Surgeons, Stamford, CT, USA; 4 Department of Intensive Care, Erasme University Hospital, Brussels, Belgium; 5 Laboratory of Experimental Intensive Care and Anesthesiology (LEICA), Academic Medical Center, University of Amsterdam, Amsterdam, the Netherlands

**Keywords:** Glucose, Spectroscopy, Point-of-care, Monitoring, ICU

## Abstract

**Background:**

OptiScanner devices, continuous glucose monitoring devices that perform automated blood draws via a central venous catheter and create plasma through centrifugation, measure plasma glucose levels through mid-infrared spectroscopy at the bedside. The objective of this study was to determine accuracy and practicality of the devices in critically ill patients attempting glycemic control.

**Methods:**

The plasma glucose level was measured by the devices and in comparative plasma samples using Yellow Springs Instrument (YSI) plasma analyzers. After adding several previously unrecognized interferences in the interference library, we reanalyzed the mid-infrared signals and compared the resulting plasma glucose level with the reference value. Results are presented in Clarke error grids, glucose prediction errors and Bland-Altman plots and expressed as correlation coefficients.

**Results:**

We analyzed 463 comparative samples from 71 patients (median 6 (4 to 9) samples per patient). After calibrating the system, a Clarke error grid showed 100% of the values in zones A or B. The glucose predictor error demonstrated that 86% of the glucose values < 75 mg/dL were within ± 15 mg/dL of the YSI results and 95% ≥ 75 mg/dL were within 20% of the comparative YSI results. Bland-Altman plot showed a bias of −0.6 with limit of agreement of −24.6 to 23.3. The Pearson correlation coefficient was 0.93 and R^2^ was 0.87. In one third of the patients the devices had to be disconnected prematurely (that is before planned disconnection) because of repeated occlusion alarms suggesting blood draw errors.

**Conclusion:**

The devices needed calibration for several previously unrecognized interferences. Thereafter, accuracy of the device to measure plasma glucose levels in ‘our cohort’ of critically ill patients improved, but external validation is highly recommended. The automated blood draw system of the devices needs further improvement to make this device of value for clinical use (trial registration (Netherlands Trial Register): NTR2864).

## Background

Critically ill patients often receive insulin infusion [1,2], although concerns with the risk of hypoglycemia and failure to show a positive impact on patient outcome has altered the frequency of use. Intense monitoring of the blood glucose level is a prerequisite for efficient and safe insulin infusion [1,2]. At present, ICU nurses monitor the blood glucose level through intermittent manually obtained blood samples for glucose measurements in central laboratories or at the bedside using different types of point-of-care analyzers [3].

Today’s blood glucose monitoring suffers from a variety of error sources that can put ICU patients at risk for insulin titration inaccuracies [4]. Moreover, intermittent glucose monitoring lacks sufficient trending, while immediate feedback and predictability of the effects of insulin could have the potential to improve titration of insulin in ICU patients [5]. Also, ICU nurses usually take > 2 mL of blood with every blood draw and the time consumed is estimated to be as high as five minutes with each draw and subsequent measurement [6], which makes intense monitoring impractical if not impossible. Automated continuous glucose monitoring (CGM) could overcome several of these problems.

OptiScanner devices (OptiScan Biomedical Corporation, Hayward, CA, USA), devices that perform automated blood draws via central venous catheters and create plasma through automatic centrifugation, measure the plasma glucose level every 15 minutes through mid-infrared spectroscopy at the bedside [7]. In a pre-clinical animal study, this device accurately measured the glucose level along hypoglycemic, euglycemic and hyperglycemic ranges [7]. Similar results came from a study with diabetic volunteers [8], and a study of frozen and stored plasma samples from critically ill patients [9]. We hypothesized that the devices provide accurate glucose level measurements in ICU patients. In this study we tested the accuracy of the devices in a cohort of critically ill patients treated according to a glucose control guideline targeting blood glucose levels between 90 and 144 mg/dL (ICU-A in [10]).

## Methods

### Study design and informed consent

This was an investigator-initiated observational study testing the accuracy of the devices in critically ill patients. OptiScan Biomedical Corporation sponsored part of the study, identified interferences and updated the interference library. The company had no influence on study design, the final analysis and the writing of this manuscript. The Institutional Review Board of the Academic Medical Center (AMC), Amsterdam, the Netherlands, approved the study protocol. Patients or next of kin had to provide written informed consent before the start of any procedure of the study. Analysis of paired data followed the analysis plan as published at [http://www.trialregister.nl/] (NTR2864).

### Study location

The study was performed in a 32-bed mixed medical-surgical ICU in a university hospital (AMC), a closed format department with patients under the direct care of an ICU team. The nurse to patient ratio is 1:2. All beds are equipped with a patient data management system (MetaVision®, iMDsoft, Tel Aviv, Israel) in which all clinical and laboratory data are stored.

### Study population

Patients were eligible for inclusion if they were aged ≥ 18 years, had an expected ICU length of stay of > three days at time of enrollment and an Acute Physiology and Chronic Health Evaluation (APACHE) II [11] score ≥ 10, had an existing arterial line, and had an existing central venous catheter (CVC) with a free lumen available. Both patients who received insulin infusion and patients who did not receive insulin infusion could be included in the study. Patients were excluded if they had received any investigational product or if they had received treatment with an investigational device within the previous 30 days or were pregnant. For practical reasons, patients in isolation for colonization with multi-resistant bacteria were excluded from participation.

ICU nurses performed glucose control with insulin, following a local guideline targeting at blood glucose levels between 90 and 144 mg/dL (ICU-A in [10]). The local guideline for glucose control advised starting continuous infusion of insulin when the blood glucose level > 144 mg/dL. Insulin was exclusively given by continuous intravenous infusion with only a bolus of insulin given when the blood glucose level exceeded > 360 mg/dL. Subcutaneous insulin is never used. Insulin infusion adjustments were made using sliding scales [10]. Insulin infusion was stopped and boluses of dextrose were given when the blood glucose level declined < 61 mg/dL. According to the local guideline for glucose control, glucose levels were to be measured at least every four hours, but for the study the study protocol dictated that comparative glucose levels were to be measured every two hours. Glucose levels used for insulin adjustment were measured in arterial blood samples using RapidLab 1265 blood gas analyzers (Siemens Healthcare Diagnostics, The Hague, the Netherlands), located in the ICU.

### OptiScanner

The device withdraws approximately 2.6 mL of blood every 15 minutes. From this, a sample of approximately 0.1 mL is extracted, heparinized, converted to plasma and analyzed by a mid-infrared spectrometer. For this, after each draw, the device centrifuges the sample, and a measurement is conducted on the plasma component using spectral analysis. This spectroscopy technique is based on the characteristic absorption of vibrational nodes of different molecules, including glucose. Mid-infrared spectroscopy can be used because the glucose spectral peaks are in the mid-infrared region [7]. After measurement, the device automatically flushes the sample to sealed waste containers, cleaning the micro-cuvette in the process. The remaining approximate 2.5 mL of blood is returned back to the patient with a small amount of saline (less than 2.0 mL). There is no heparin in the blood that is returned to the patient. The devices have an onboard interference library which includes drugs and endogenous substances that is used to subtract out interferences.

### Study procedures

One device was connected to the proximal port of an existing CVC for up to 24 hours in the first 65 patients, and for up to 72 hours in the last 10 patients. Every 15 minutes, the device withdrew blood and measured the plasma glucose level. These results were not visible for ICU nurses and staff, but were downloaded to a computer after disconnection of the device. Comparative arterial blood draws were taken every two hours, at the same time as the device was drawing venous blood, starting from approximately 8 am until 10 pm. Comparative glucose levels in plasma were measured immediately with the YSI 2300 STAT Plus (Yellow Springs Instrument, Yellow Springs, OH, USA) and with the RapidLab 1265 blood gas analyzers (Siemens Healthcare Diagnostics, The Hague, the Netherlands). The results of the blood gas analyzers were available for the nurses for insulin titration.

### Dealing with new interfering substances

An interfering substance was suspected if we found significant discrepancies between paired fixed-wavelength mid-infrared glucose measurements and YSI readings. OptiScan Biomedical Corporation then analyzed the source documentation to identify what substances could have affected the spectra. Pure spectra of the identified interferences were then isolated and subsequently proven to interfere in separate laboratory experimentation by OptiScan Biomedical Corporation. If subsequently verified, the interference was added to the interference library. First, the updated interference library was tested in the already collected spectroscopies. Thereafter, the interference library was updated to the onboard database and used in subsequent patients.

The onboard interference library was updated twice during the study, that is after inclusion and analysis of patient 28 and patient 63, and once after inclusion of the last patient. We thus had accrued three different interference libraries during the study, and one new interference library at the end of the study. After adding all validated interferences in the interference library, the mid-infrared spectroscopies were reanalyzed by OptiScan Biomedical Corporation, calculating a new glucose value for every collected spectroscopy.

### Statistical analysis

**Figure 1:**
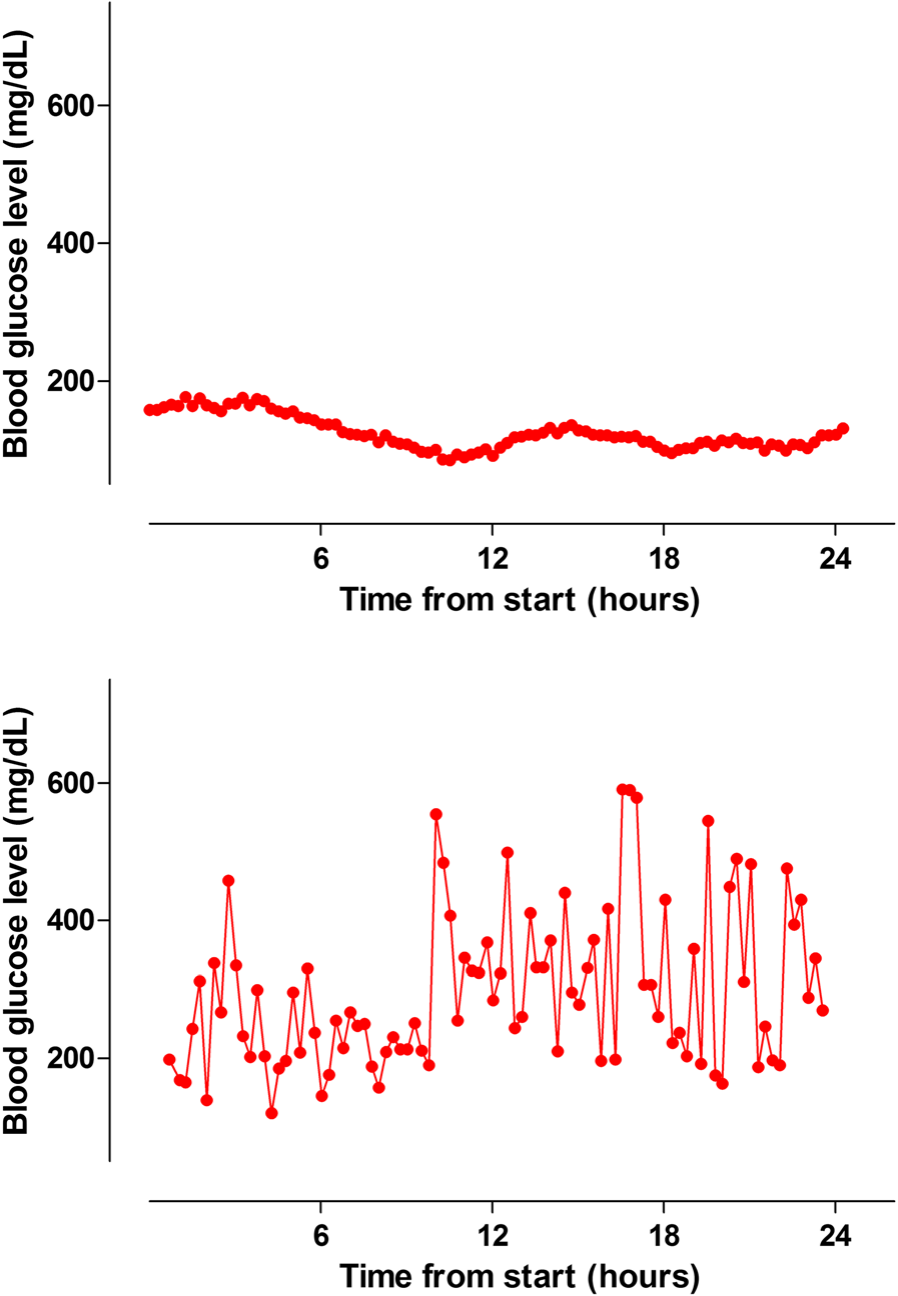
An example of a patient without interferences (upper panel) and of a patient with non-physiological glucose changes caused by simultaneous infusion of parenteral nutrition (lower panel).

In two patients, we detected frequent non-physiological glucose changes, defined as increases and/or decreases of the glucose level more than 80 mg/dL in consecutive device-readings while insulin infusion rates were not altered. These non-physiological glucose changes were caused by simultaneous intravenous infusion of 5% dextrose in one patient, and parenteral nutrition in another patient via another lumen of the CVC. Therefore these two patients were excluded for analysis. Figure 1 shows an example of a patient without interferences and a patient with non-physiological glucose changes in a patient who received parenteral nutrition via another lumen of the CVC used for sampling by the device.

We report data as means (± SD) or medians (IQR) where appropriate. In order to be considered for the statistical analysis, each patient needed to have at least two consecutive and successfully analyzed blood draws.

Clarke error grid (CEG) analyses were used to show the percentage of paired values falling within predefined zones. The CEG is divided in ten zones: in the two zones A the device results are < 20% of the YSI results, which is seen as clinical acceptable, or the results are < 70 mg/dL; in the two zones B the device results deviate > 20% from the YSI result, but deviations are seen as clinically acceptable because they are considered only to cause benign insulin titration errors; in the zones C to E the deviations become increasingly unacceptable; in zones D potentially dangerous hypoglycemia or hyperglycemia are left undetected, and in zones E device results lead to opposite treatment decisions, that is the device suggests hypoglycemia while the YSI shows hyperglycemia, or *vice versa*[12].

For each pair of device and YSI results, the glucose prediction error was defined as (device result – YSI result). The percentage of paired data points with glucose prediction errors are presented according to the current International Standards Organization standard (ISO15197), with the percentage of data points specified that fall within ± 15 mg/dL of the YSI results for YSI glucose level of < 75 mg/dL, and within ± 20% of the YSI results for YSI glucose level ≥ 75 mg/dL. The higher the percentages, the better accuracy for YSI results < 75 mg/dL, or ≥ 75 mg/dL.

Bland-Altman plot with bias (mean difference between the device and YSI measurements) and limits of agreement (bias ± 1.96 x standard deviation of the bias) were used to show agreement between the device and YSI measurements [13]. The limit of agreement shows the band in which 95% of the device results deviate from the mean difference between the YSI and device results. The smaller the bias and limit of agreement, the better agreement between the YSI and device results [13].

The results of the device were regressed on the results of the YSI using unweighted least squares. The linearity between the device and the YSI over the study measurement range were assessed by the Pearson correlation coefficient and coefficient of determination, R^2^.

To assess if the occurrence of occlusion alarms was related to the insertion site of the CVC, patients were divided into groups based on the location of their CVC (jugular, femoral or subclavian). The Chi-square test was used to compare the different locations with and without alarms. A *P-*value < 0.05 was considered statistically significant.

Analyses were performed using IBM SPSS Statistics 20 (IBM, Armonk, New York, United States). R (version: 2.15.1; R Foundation for Statistical Computing, Vienna, Austria) was used for CEG, glucose prediction error, Bland-Altman plot and linearity tests.

## Results

### Patients

**Figure 2:**
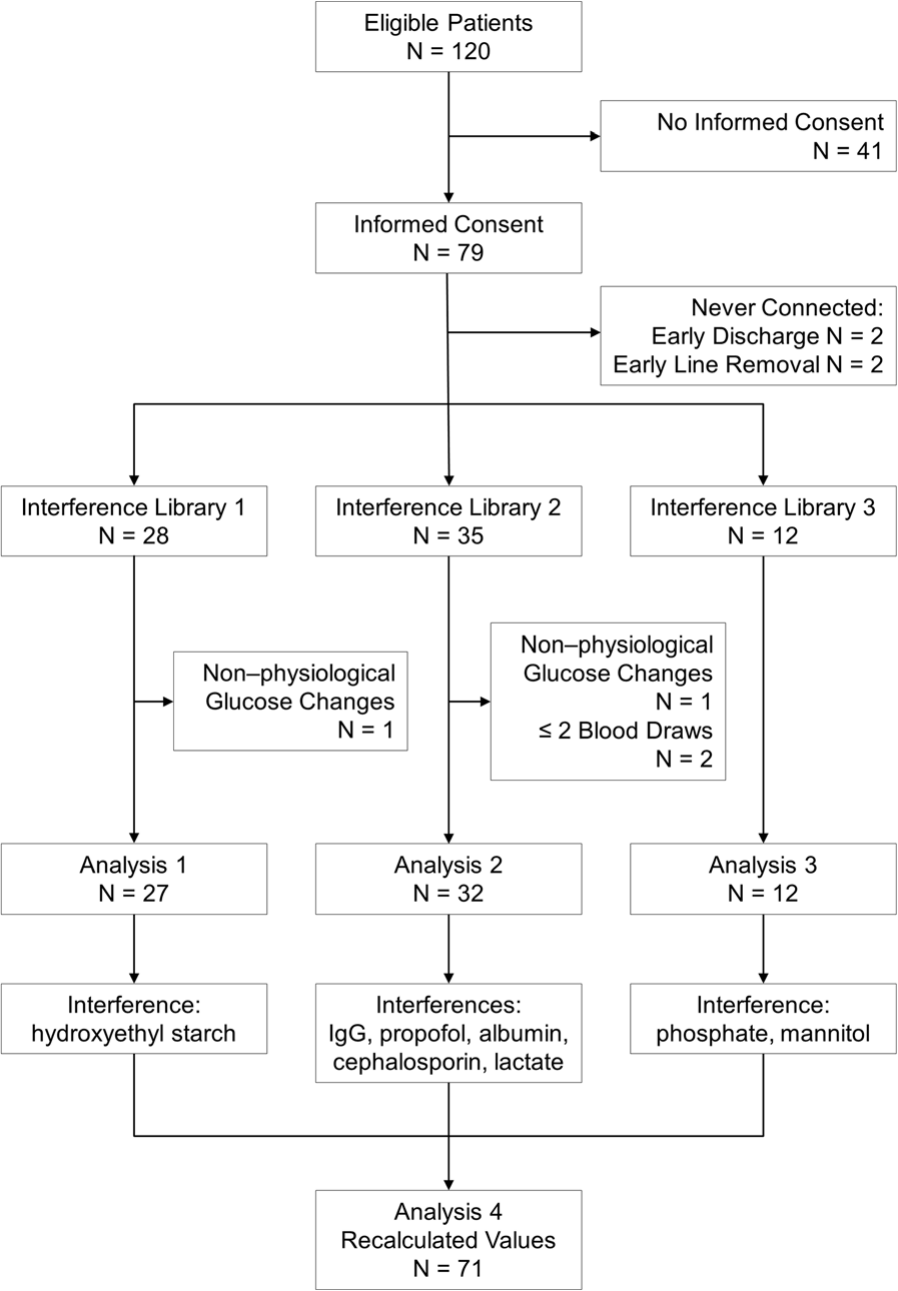
CONSORT diagram of the study.

We included 75 patients (Figure 2). Two patients from whom non-physiological blood glucose changes were noted and two patients, from whom we could not collect the minimum number of two consecutive blood draws, were excluded from the analysis.

Patient characteristics and metrics of glucose control are shown in Tables 1 and 2. The median connection time was 22.9 (11.4 to 24) hours for patients planned to be connected up to 24 hours and 28.4 (3.2 to 63.3) for the final ten patients planned to be connect up to 72 hours. In total, we used 101 cartridges (median 1 (1 to 2) per patient planned to be connected up to 24 hours; median 1 (1 to 2.25) per patient connected up to 72 hours).

**Table 1:** Patient characteristics

Age (years), median (IQR)	65 (55 to 73)
Male gender, number (%)	44 (62)
Admission diagnosis, number (%)	
Medical	42 (59.2)
Surgical	18 (40.8)
Unplanned admission	56 (78.9)
APACHE II scores, median (IQR)_	21 (16 to 26)
SAPS II, median (IQR)	43 (36 to 54)
ICU LOS (days), median (IQR)	10 (5 to 20)
Hospital LOS (days), median (IQR)	20 (12 to 34)
ICU mortality, number (%)	16 (22.5)
Hospital mortality, number (%)	20 (28.2)
Central venous catheter insertion site, number (%)	
Femoral	30 (42.3)
Jugular	30 (42.3)
Subclavian	11 (15.5)
Type of central venous catheter, number (%)	
Trilumen	23 (32.4)
Quad lumen	48 (67.6)

*Abbreviations*: *APACHE* Acute Physiology and Chronic Health Evaluation, *BMI* body-mass index, *ICU* intensive care unit, *IQR* interquartile range, *LOS* length of stay, *SAPS* Simplified Acute Physiology Score.

**Table 2:** Measures of blood glucose control

Number of measurements	484
Blood glucose level - mg/dL (median (IQR))	130.3 (113.0 to 147.3)
Blood glucose level - mg/dL (mean ± SD)	134.3 ± 35.0
Number of measurements per patient (median, IQR)	6 (4 to 9)
Severe hypoglycemia < 40 mg/dL - measurements, number (%)	0
Severe hypoglycemia < 40 mg/dL - patients, number (%)	0
Mild hypoglycemia < 75 mg/dL - measurements, number (%)	8 (1.7%)
Mild hypoglycemia < 75 mg/dL - patients, number (%)	5 (7.0%)
Mild hyperglycemia > 150 mg/dL - measurements, number (%)	110 (22.7%)
Mild hyperglycemia > 150 mg/dL - patients, number (%)	36 (50.7%)
Severe hyperglycemia > 180 mg/dL - measurements, number (%)	42 (8.7%)
Severe hyperglycemia > 180 mg/dL - patients, number (%)	13 (18.3%)

Data consider the period of the study to be from when the OptiScanner® was connected.

### Accuracy of devices in critically ill patients

**Figure 3:**
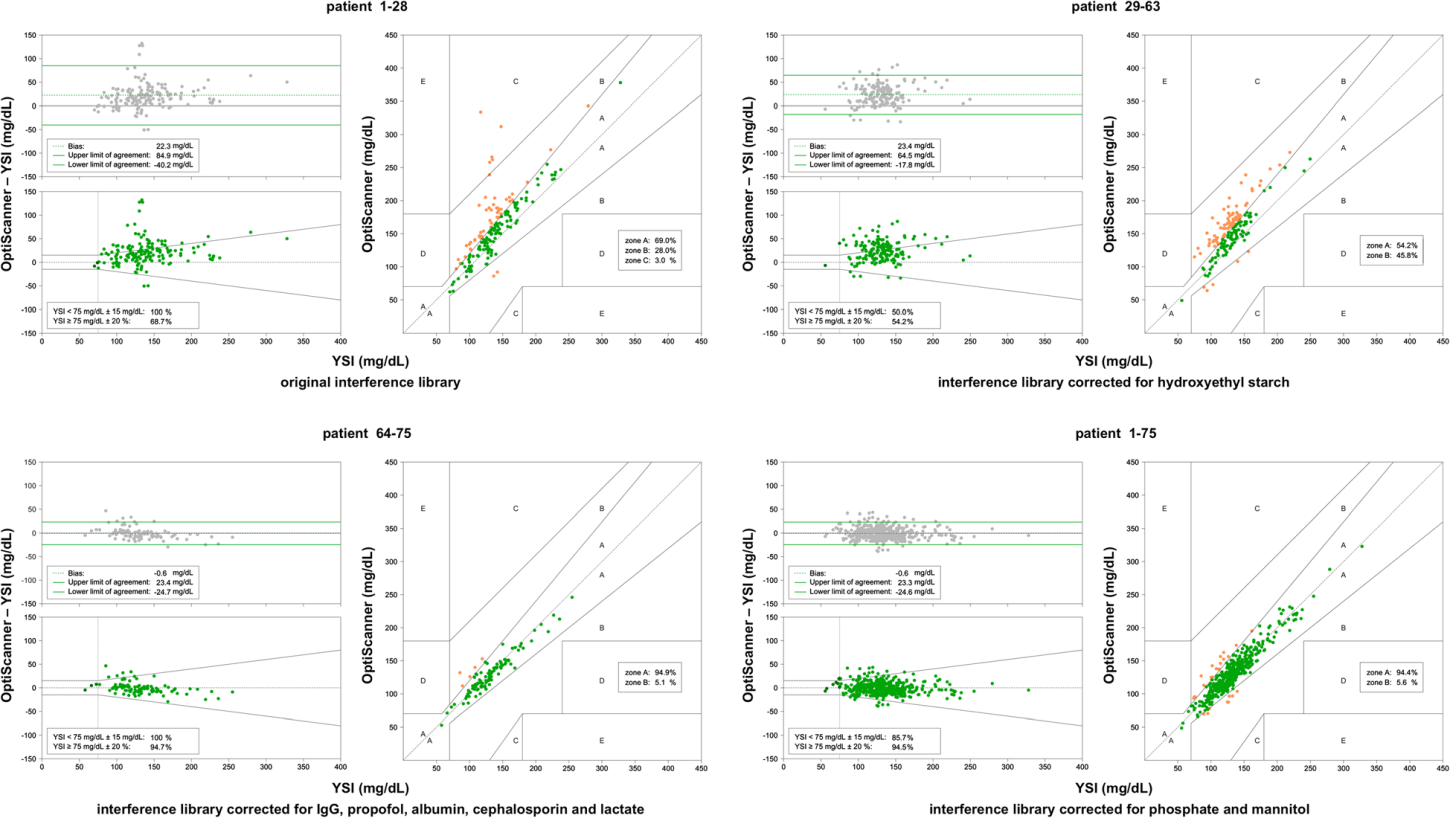
**Bland-Altman plot (upper left panels), glucose prediction error (left lower panels) and Clarke error grid (right panels) for patients 1 to 28 using interference library 1 (left upper box), for patients 29 to 63 using interference library 2 (right upper box) and patients 64 to 75 using interference library 3 (left lower box).** The paired samples of all patients together (that is, patients 1 – 28 + 29 – 63 + 64 –75) were recalculated using interference library 4 (right lower box).

We collected 168 paired samples in 27 patients using interference library 1, 168 paired samples in 32 patients using interference library 2, and 98 paired samples in 12 patients using interference library 3. CEGs (Figure 3), glucose predictor error (Figure 3) and Bland-Altman plot (Figure 3) demonstrated that the accuracy of the device improved with the two updates of the interference libraries. Indeed, the percentage of paired samples in zones B and C of the CEG decreased with successive updates. In accordance, the glucose prediction errors and Bland-Altman plots showed an improved accuracy and a smaller bias and limits of agreement. The Pearson correlation coefficient and R^2^ increased from 0.80 and 0.63 (with interference library 1), to 0.82 and 0.67 (with interference library 2) and 0.93 and 0.88 (with interference library 3).

With the recalculated values we analyzed all paired samples (Figure 3). The correlation coefficient was 0.93 and R^2^ was 0.87. The accuracy of the results of the final 12 patients was consistent with the reanalysis by OptiScan Biomedical Corporation of the overall study population.

### New interferences

We found the following interferences; 6% hydroxyethyl starch (Venofundin®, B Braun, Melsungen, Germany); 20% human albumin (Albuman®, Sanquin, Amsterdam, the Netherlands, immunoglobulin G (Nanogam®, Sanquin, Amsterdam, the Netherlands), propofol (Propofol-®Lipuro, B Braun, Melsungen, Germany), cephalosporin (including cefotaxim and ceftazidim), lactate (infusion fluid containing lactate used for continuous veno-venous hemofiltration), mannitol and infusion of phosphate (Figure 1).

### Practicality of the devices in critically ill patients

The device was prematurely disconnected (that is, before 24 hours in the first 65 patients, or before 72 hours in the remaining 10 patients) in 8 patients because of early patient discharge, death, or the necessity to use the lumen of the CVC for infusion of medication, and in 26 patients because of device errors (two patients) or problems with withdrawal of blood from the central venous line. In 32 patients occlusion alarms occurred, suggesting that the devices could not draw blood from the CVC (Table 3). In eight patients this alarm was solved through flushing the connection system. In 24 patients, this alarm could not be solved and the device was disconnected. The occurrence of occlusion alarms was independent of the insertion site of the CVC (femoral versus jugular versus subclavian) or type of CVC (trilumen versus quad lumen).

**Table 3:** Occlusion alarms

	**Number (%) of**	**Number (%) of**
	**patients**	**cartridges**
Total alarms	32 (45%)	36 (36%)
Per insertion site		
Femoral	14 (47%)	17 (42%)
Jugular	12 (40%)	13 (30%)
Subclavian	6 (55%)	6 (35%)
Per type of central venous catheter		
Trilumen	14 (61%)	15 (52%)
Quad lumen	18 (38%)	21 (29%)
Alarms leading to disconnection	24 (34%)	24 (24%)
Per insertion site		
Femoral	12 (40%)	12 (29%)
Jugular	8 (27%)	8 (19%)
Subclavian	4 (37%)	4 (24%)
Per type of central venous catheter		
Trilumen	11 (48%)	11 (38%)
Quad lumen	13 (27%)	13 (18%)

In 18 out of 101 (18%) cartridges an error occurred, mandating replacement of the cartridge. Twelve cartridge failures occurred prior to connecting the device to the patient, that is during the systems initial self-diagnostic check of the system. After connection, in four cases there was a problem with the fluidics system within the cartridge; in one case blood could not be drawn and in one case there was a problem with loading heparin into the cartridge. A system error, mandating a reset of the system, occurred three times. In all cases this happened before connection of the device to the patient. We could not further determine what actually happened, because the logging capabilities failed to analyze this error.

## Discussion

The present study shows that the device, after calibration for previously unrecognized interferences, had a better accuracy for measuring plasma glucose levels in critically ill patients. However, in a significant number of patients the devices had to be disconnected prematurely because of occlusion alarms, which could not easily be overcome by flushing the system.

The results of the present investigation confirm findings from previous studies with the same devices. One study of otherwise healthy diabetic patients showed 99% of data in zones A or B of the CEG analysis [8]. Another study testing *ex vivo* point accuracy of the same devices using frozen plasma samples from a series of ICU patients found a correlation coefficient of 0.94 [9]. While the device was designed for critically ill patients it had not yet been tested *in vivo*, and specifically, in critically ill patients at the ICU bedside. The present study expands the experience with the devices and shows that the devices could perform accurate glucose level measurements.

The accuracy of the device improved after adding interferences to the interference library. Indeed, after adding the interferences as identified in this study cohort, all paired samples were in zones A and B.

The total percentage of paired data values outside the glucose prediction error of the device is around 6%, less accurate than most of the blood gas analyzers [14]. Several different continuous glucose monitoring devices are being developed, using dissimilar sampling techniques and sensors [1]. The reports on accuracy of other continuous glucose monitoring devices show comparable accuracies, with 95 to 100% of the paired measurements in zone A and B of the CEG [12,13,15-18].

Even though numerous interferences were already identified in previous studies [7-9], several new ones were identified. We were not surprised to find interferences, although we did not expect to find such commonly used substances as interferences. This is probably explained by the fact that the devices were previously only *ex vivo* tested in an ICU patient cohort. Furthermore, this cohort was in another continent, where other medications and infusion fluids are used [10]. The finding of commonly used medications as interferences shows the importance of external validation in different continents and countries, and maybe even hospitals. Therefore, caution is needed with the implementation of devices.

Additionally, we encountered cartridge errors, and system errors which occurred before connection of the device to the patient. We had to disconnect the device frequently because of occlusion alarms, suggesting that the device could not draw a blood sample. Sometimes occlusion alarms could be easily overcome by flushing the connection system. If not, manipulation of the CVC was needed to facilitate blood draws. We are uncertain whether these problems may persist in daily clinical practice.

Notably, results from two patients with simultaneous glucose infusion via another port of the CVC were excluded in the analysis. While simultaneous glucose infusion via the same infusion system that is used for measuring glucose levels should be avoided, the finding clearly shows the risk of erroneous readings.

Any device that uses a CVC has the potential to increase the risk of catheter-associated blood stream infections, especially when blood is retracted repeatedly through the CVC. In addition, the latter could also cause clotting in the lumen. We explicitly looked for these clinically important complications but did not find them in this cohort of patients. It should be noted, though, that this study is too small to draw firm conclusions regarding the risk of blood stream infections or clotting. Also, we did not find a difference in occurrence of occlusion alarms between insertion site or type of catheters. In some cases of occlusion, alarms were solved by manipulation of the CVC. It should be noted, though, that the need for manipulation of the CVC could be a shortcoming of the device. Second, it should be noted that nurses were allowed to disconnect the device at any moment an occlusion alarm occurred in the absence of the investigators. However, it is unknown what happens if repeated occlusion alarms occur and are resolved, that is whether this promotes clotting of the lumen. This needs to be investigated in future studies.

One limitation of the study is that we only collected paired glucose measurements during working hours, which could have prevented us from finding additional interferences (for example, medications that are only infused during the night). Furthermore, this was a single-center study. Also, it is possible that additional interferences could be found when the devices are used in other patient populations.

In addition, the number of paired glucose measurements per patient was small. An additional limitation of the study is that most data points were in a narrow glucose range - we only had 8 of the 484 paired measurements < 75 mg/dL and 42 of the 484 paired measurements > 180 mg/dL. We did not have the opportunity to assess the ability of the device to detect severe hypoglycemia (that is, < 40 mg/dL) as it did not occur during conduct of the study. Here, the low incidence of hypoglycemia suggests that the nurses were experts in insulin titration. Indeed, the nurses had experience with the insulin infusion guideline for many years. In addition, the blood glucose target in the local guideline is higher compared to that in the original Leuven studies, which could also have affected the incidence of severe hypoglycemia. Furthermore, because nurses were aware that the devices monitored the blood glucose level in their patients frequently, it could be that they titrated insulin more precisely. Finally, the nurses had access to all blood glucose levels, thus also the extra ones taken for the study. As such, nurses could also better prevent severe hypoglycemia.

## Conclusion

The blood glucose measuring devices we used needed calibration for several previously unrecognized interferences. Thereafter, accuracy of the device for measuring plasma glucose levels in ‘our cohort’ of critically ill patients improved, but external validation is highly recommended. Also, the automated blood draw system of these devices needs further improvement to make them of value for clinical use.

## Abbreviations

AMC: Academic Medical Center; APACHE: Acute Physiology and Chronic Health Evaluation; BMI: body-mass index; CEG: Clarke error grid; CGM: continuous glucose monitoring; CVC: central venous catheter; IQR: interquartile range; LOS: length of stay; SAPS: Simplified Acute Physiology Score; SD: standard deviation; YSI: Yellow Springs Instrument.

## Competing interest

Roosmarijn TM van Hooijdonk reported consulting work for Medtronic Inc., GlySure Ltd and research support from Medtronic Inc. and OptiScan Biomedical. Tineke Winters reported consulting work for Medtronic Inc., GlySure Ltd and OptiScan Biomedical. James S Krinsley reports receiving consultant fees from Medtronic Inc., Edwards Life Sciences, Roche Diagnostics, OptiScan Biomedical and Alere and research support from OptiScan Biomedical. Jean-Charles Preiser reported receiving consultant fees from Medtronic Inc., Edwards Life Sciences and OptiScan Biomedical. Johan C Fischer and Edmée C van Dongen-Lases reported no relevant disclosures. Marcus J Schultz reported receiving consultant fees from Medtronic Inc., GlySure Ltd., Edwards Life Sciences and Roche Diagnostics and financial support from Medtronic Inc. and OptiScan Biomedical - all fees and financial supports were paid to the institution. The AMC Medical Research BV received €60.000 for covering personnel and laboratory costs associated with performing this trial. OptiScan Biomedical provided the OptiScanner devices and cartridges free of charge. The investigators were free to make a publication and had no restrictions made by OptiScan Biomedical. OptiScan Biomedical was only allowed to check the publication for company proprietary information. The authors declare that they have no competing interests.

## Authors’ contributions

Study concept and design: RTMvH, TW, JCF, ECD-L and MJS. Acquisition of data: RTMvH, TW and MJS. Analysis and interpretation of data: RTMvH and MJS. Drafting of the manuscript: RTMvH and MJS. Critical revision of the manuscript for important intellectual content: RTMvH, TW, JCF, ECvD-L, JSK, J-CP and MJS. All authors read and approved the final manuscript.
